# Supplementary material for: Reweighting of molecular simulations with explicit-solvent SAXS restraints elucidates ion-dependent RNA ensembles
Source: Nucleic Acids Res. 2021 Jun 9;49(14):e84. doi: 10.1093/nar/gkab459 (PMC8373061; doi:10.1093/nar/gkab459)
Supplement: gkab459_Supplemental_File [file gkab459_supplemental_file.pdf]

Supporting Information for: Reweighting of molecular simulations with explicit-solvent SAXS restraints elucidates ion-dependent RNA ensembles

Mattia Bernetti, Kathleen B. Hall, and Giovanni Bussi

May 7, 2021

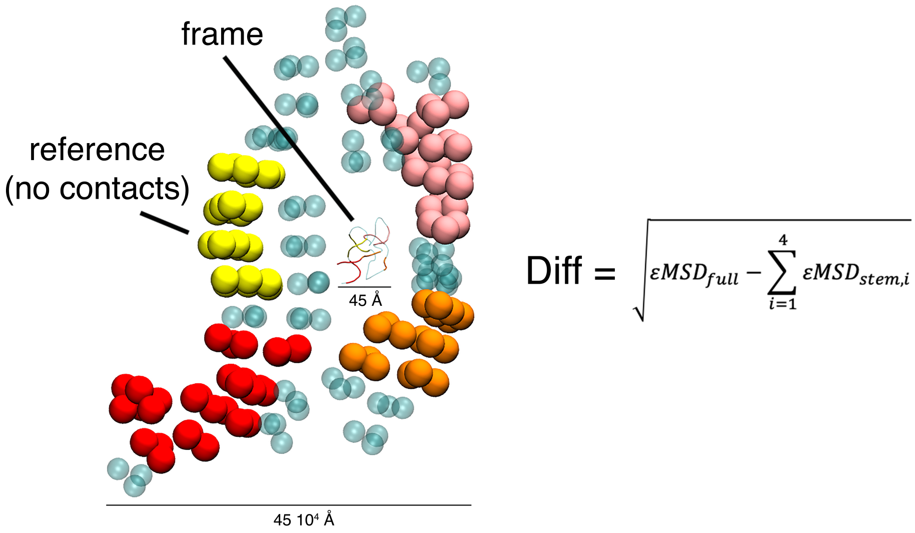

Figure S1: Schematic depiction of the Diff CV employed in the metadynamics enhanced-sampling MD simulations. For the RNA structure of an MD frame, the CV variable is defined as the root mean square of the G-vectors and can be computed in practice taking the root-square difference of the eRMSD [1] with respect to an arbitrary structure with no contacts formed (reference, no contacts), computed using either the whole sequence (full) or only the stem regions (stem). The heavy atoms of the structure with no contacts formed, represented here as spheres, are color coded consistently with Figure 2 of the main text.

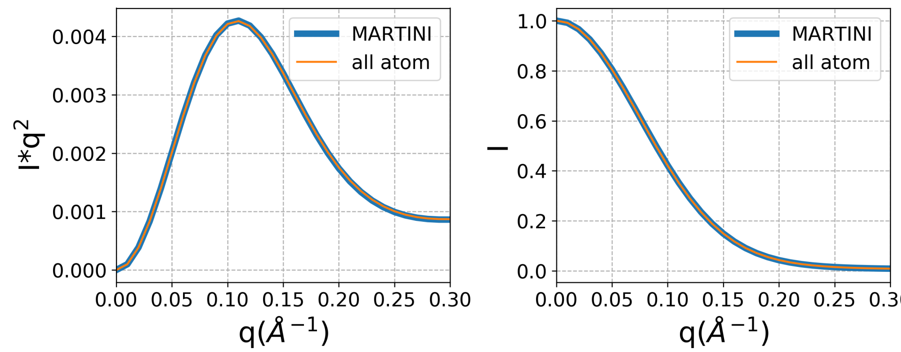

Figure S2: Sample pure-solute SAXS spectra computed through PLUMED from the all-atom RNA structure (orange line) or relying on a MARTINI bead representation of the structure (blue line). The SAXS spectra are shown both in the $I * q^2$ vs q Kratky form (left panel) and in the classical I vs q form.

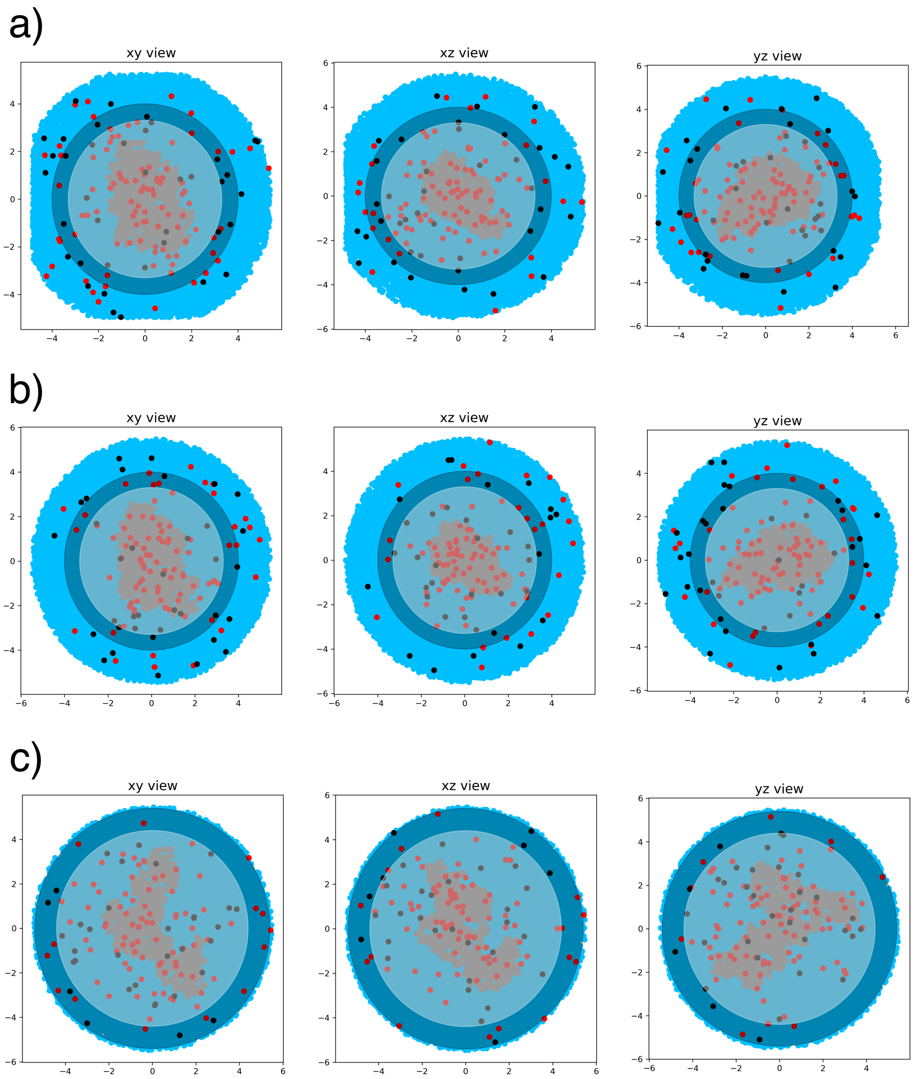

Figure S3: Schematic depiction of sphere radiuses and shell widths employed for the solvent matching in the computation of the SAXS spectra through the Capriqorn software for a) the long unbiased MD simulations, b) structures extracted from the metadynamics simulations having R_g compatible with the unbiased simulations, and c) structures extracted from the metadynamics simulations having the highest value of R_g, *i.e.*, the most extended structures sampled. Each panel gives the projection of the system coordinates along two specified axes, with water oxygens shown in sky blue, K^+ ions in red, Cl^- in black, and RNA heavy atoms in gray. The shell width and radius used by Capriqorn are displayed as dark shade and sum of black shade and white shade, respectively.

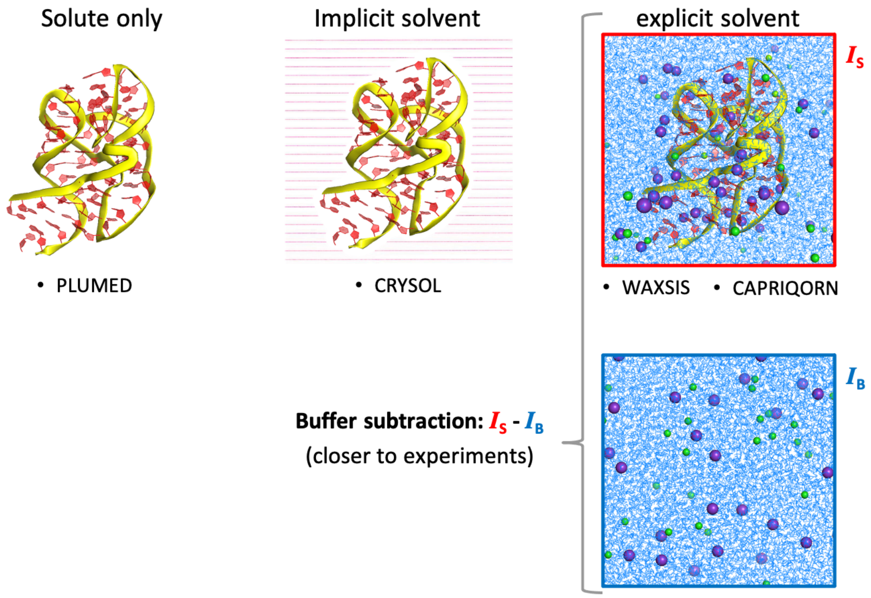

Figure S4: Schematic comparison of different available strategies for including the solvent contribution in the computation of the SAXS spectra. Pure solute strategies (left panel) employ the solute coordinates only; a correction to account for the displaced solvent volume can be included, as implemented in PLUMED. Implicit solvent strategies (central panel), as implemented in the CRYSOL software, add a term for the solvent contribution due to the hydration shell around the solute's surface. Conversely, explicit solvent strategies (right panels), as in the WAXSiS and Capriqorn software, include explicitly all the system's atoms (solute + solvent) in the computation of the SAXS spectra. Notably, in the latter procedure the final spectrum is obtained from the difference of two separate simulations, one of whole system (solute + solvent) and one of the solvent only. Such practice is the closest to the experimental one, where the SAXS spectrum is taken from the difference of two separate measurements, one with solute and solvent and one with the solvent alone.

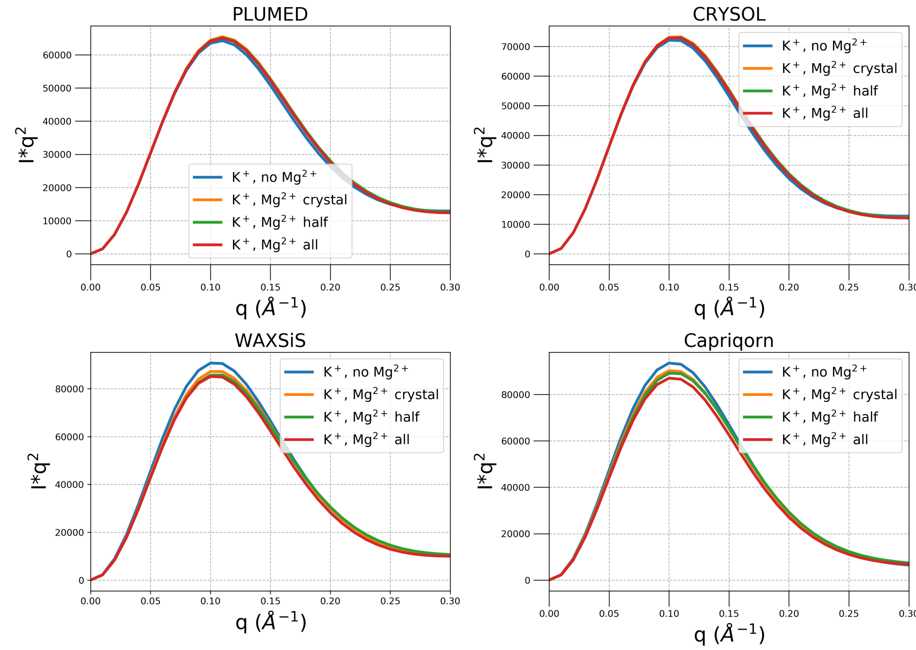

Figure S5: SAXS spectra in the Kratky form for the four systems displayed in Figure 3a of the main text. Four different software to compute the spectra are compared, namely PLUMED, CRYSOL, WAXSiS and Capriqorn.

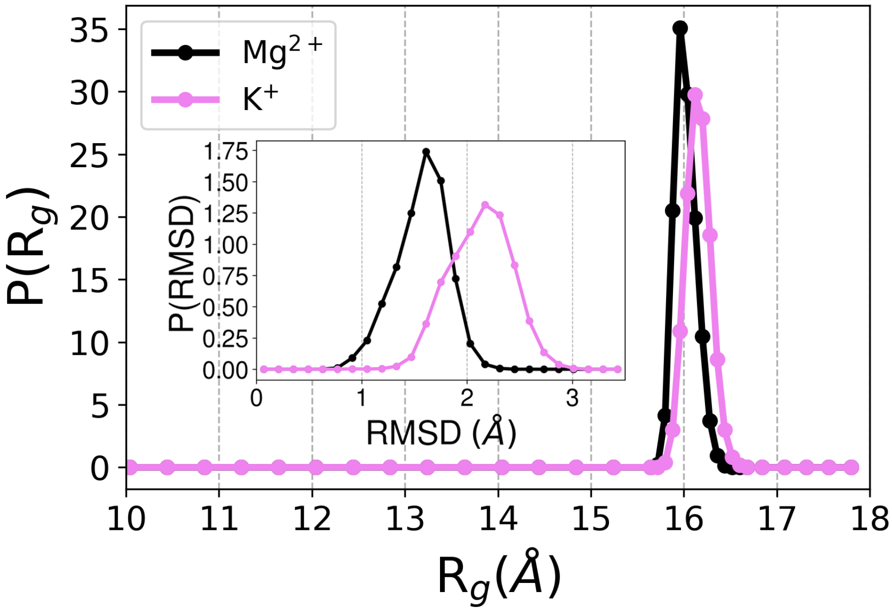

Figure S6: Probability density function of the geometric gyration radius R_g and RMSD from the starting structure for the two 1 μs long unbiased MD simulations, performed respectively on systems 1 (here labelled K^+) and 4 (here labelled Mg^{2+}) depicted in Figure 3a of the main text.

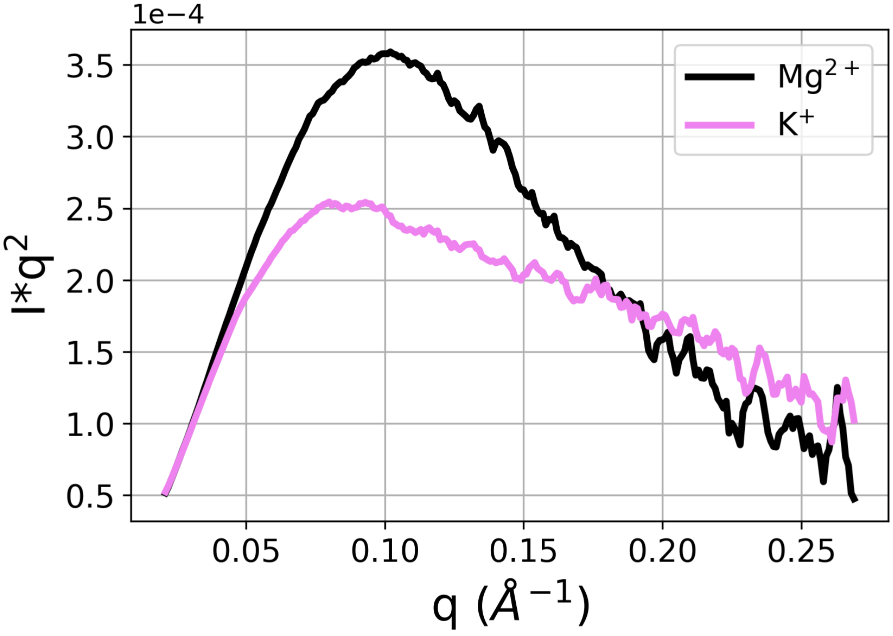

Figure S7: Reference experimental SAXS spectra, as obtained in a previous work on GAC RNA [2].

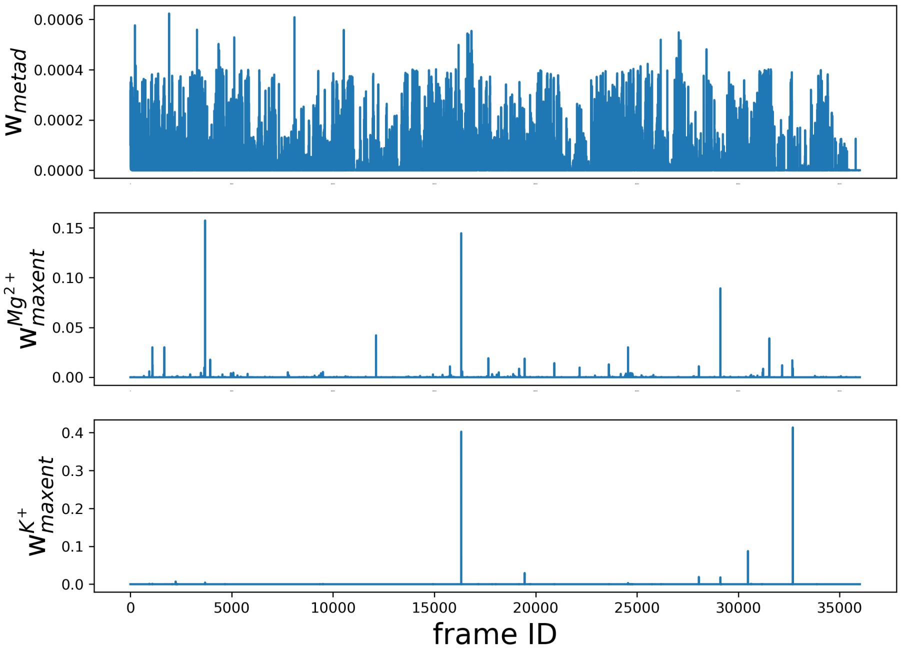

Figure S8: Weights assigned to the trajectory frames obtained from the enhanced sampling simulation. The original metadynamics weights (prior) are displayed in the top panel, while the weights resulting from reweighting through maximum entropy (posterior, refined ensembles) are shown in the central and bottom panels. In particular, the weights obtained enforcing the SAXS reference experimental data measured in presence of Mg^{2+} (central panel) and K^+ (bottom panel) and using the SAXS spectra computed for the whole system (solute + solvent) with Capriqorn as input observables, are reported.

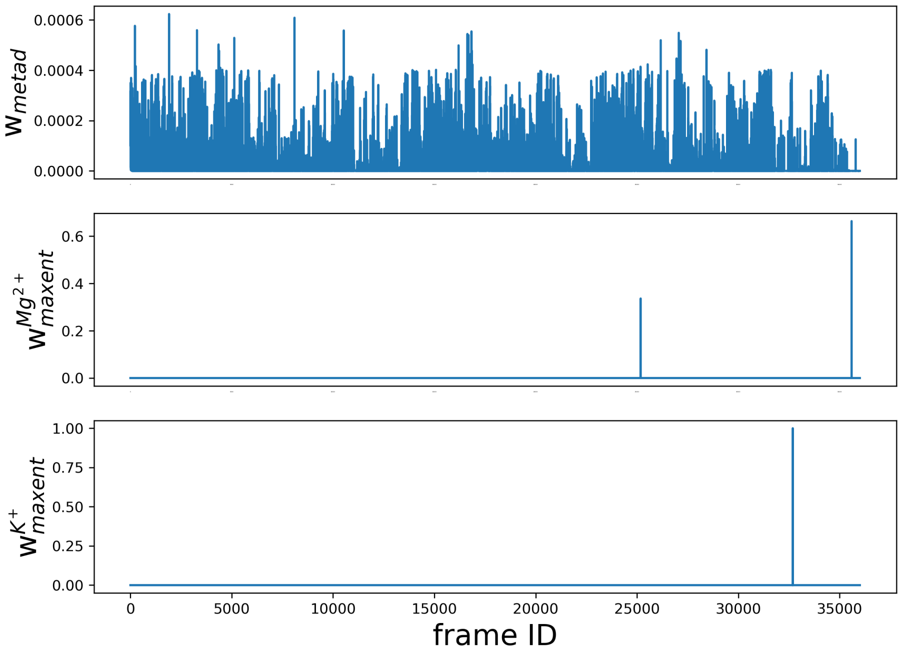

Figure S9: Weights assigned to the trajectory frames obtained from the enhanced sampling simulation. The original metadynamics weights (prior) are displayed in the top panel, while the weights resulting from reweighting through maximum entropy (posterior, refined ensembles) are shown in the central and bottom panels. In particular, the weights obtained enforcing the SAXS reference experimental data measured in presence of Mg^{2+} (central panel) and K^{+} (bottom panel) and using the SAXS spectra computed for the pure solute system with PLUMED as input observables, are reported.

a)

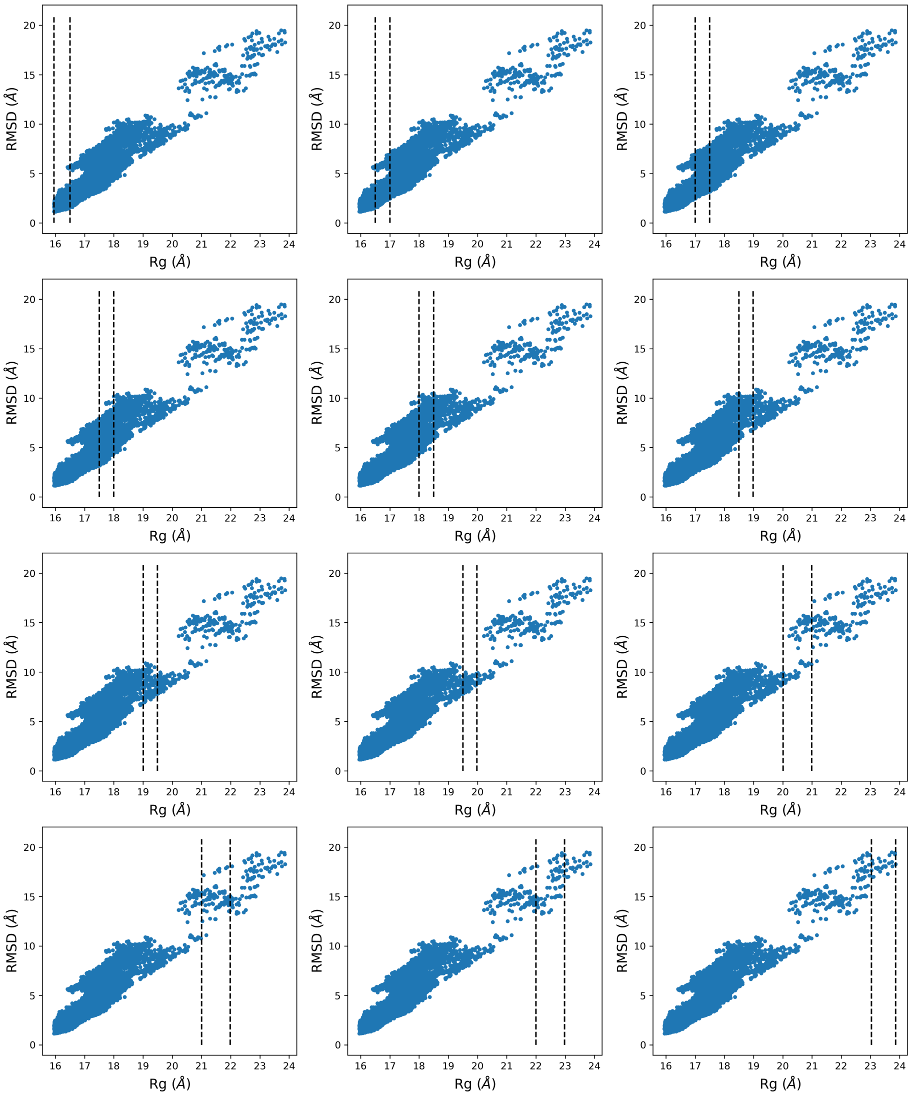

b)

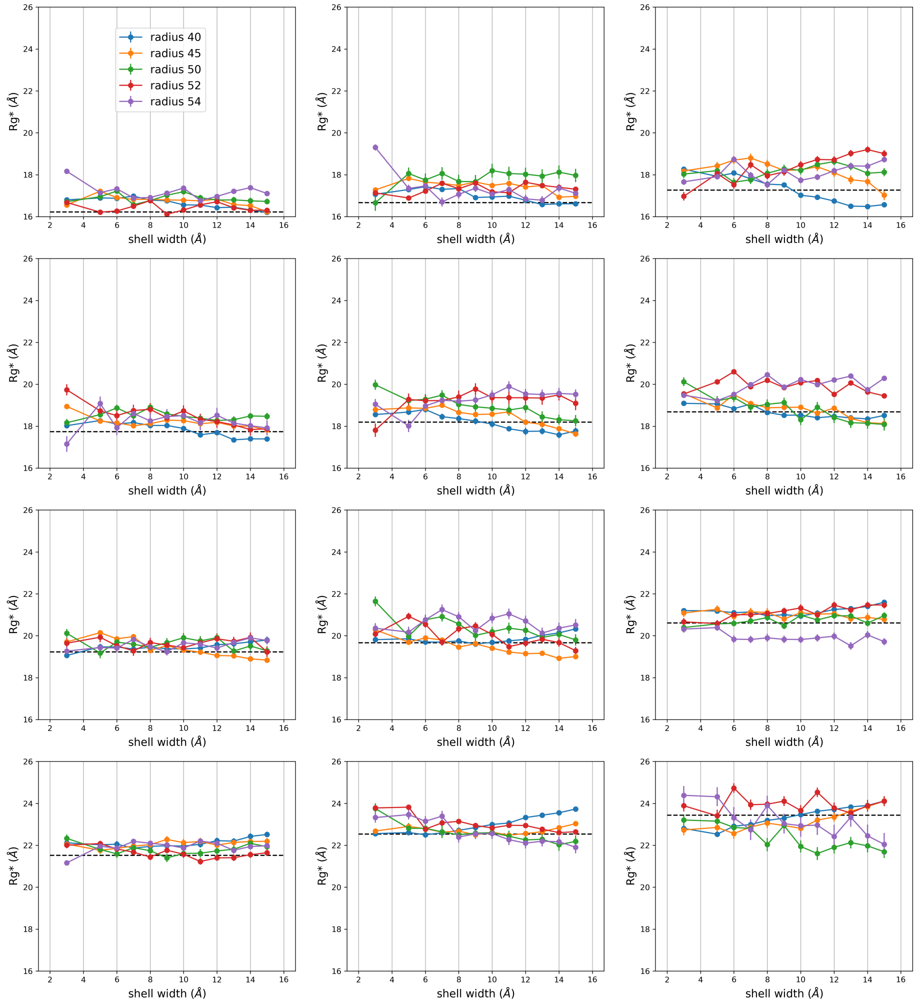

Figure S10: Computation of R_g^* for varying values of the sphere radius and shell width parameters employed for the solvent matching of the Capriqorn software. The R_g^* were computed from pools of MD frames at decreasing structural compactness extracted from the MD trajectory resulting from the enhanced sampling simulation. In particular, a) the frames were extracted from different windows (delimited by the two vertical dashed lines) at increasing values of the R_g computed from the coordinates; each considered window is displayed in the space defined by R_g from the coordinates vs RMSD with respect to the initial structure of the simulation, were all the trajectory frames are projected (blue points). For each window, b) the corresponding values of R_g^* computed through Guinier fit on the SAXS spectra computed with Capriqorn at varying values of the sphere radius and shell width parameters are shown; the average value of the R_g computed from the coordinates for that window is also displayed as horizontal dashed line.

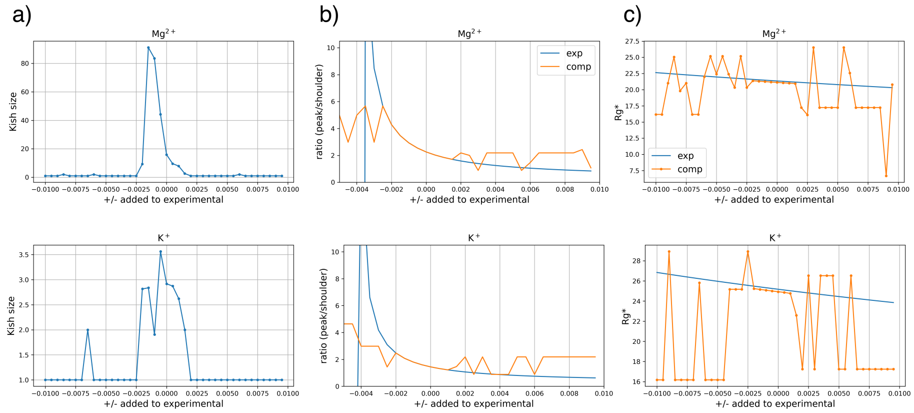

Figure S11: Effect of applying an offset to the experimental data. Reweighting through the maximum entropy principle was conducted by enforcing experimental SAXS data after a shift was added to the experimental spectra. The offset was applied to explore the effect of possible mismatched buffers in the experiment. Specifically, evenly spaced values in the range from -0.01 to +0.01 were considered. The effect on a) the Kish's effective sample size, b) the ratio (peak/shoulder of the spectra in the Kratky form) and c) R_g^* (computed via Guinier fit as described in the methods section of the main text) from the reweighted ensembles for Mg^{2+} (top panels) and K^+ (bottom panels) are reported in the figure. The Kish size is maximized for a shift that is close to zero in both cases. In particular, the Rg* obtained using the shift that maximizes the Kish size is very close to the R_g^* obtained from the unshifted spectra. This confirms that it is not necessary to add a shift to the experimental data points.

	Ratio_{exp}	$R^*_{g,exp}$	Ratio_{solv}	$R^*_{g,solv}$	Ratio_{solute}	$R^*_{g,solute}$
Mg^{2+}	2.25	21.35	2.26	21.12	1.62	21.17
K^+	1.43	25.17	1.44	24.92	0.67	22.54

Table S1: Comparison of reweighted spectra and experimental reference. Ratio, i.e. intensity of the peak (q=0.1 Å^{-1}) over shoulder (q=0.2 Å^{-1}) in the Kratky form of the spectra, and radius of gyration from Guinier fit (R^*_g) were computed from the experimental SAXS spectra (Ratio_{exp}, $R^*_{g,exp}$) and from the reweigthed spectra including the solvent contribution (Ratio_{solv}, $R^*_{g,solv}$) or the pure solute only (Ratio_{solute}, $R^*_{g,solute}$).

References

[1] S. Bottaro, P. Banáš, J. Šponer, and G. Bussi, "Free energy landscape of gaga and uucg rna tetraloops," *J. Phys. Chem. Lett.*, vol. 7, no. 20, pp. 4032–4038, 2016.

[2] R. Welty, S. A. Pabit, A. M. Katz, G. D. Calvey, L. Pollack, and K. B. Hall, "Divalent ions tune the kinetics of a bacterial gtpase center rrna folding transition from secondary to tertiary structure," *RNA*, vol. 24, no. 12, pp. 1828–1838, 2018.
